# Two Breasts, Two Malignancies: Discordant Bilateral Breast Cancers With Brain and Bone Metastases

**DOI:** 10.7759/cureus.118015

**Published:** 2026-10-16

**Authors:** Dhanvi Patel, Nidhi Patel, Shreya Patel

**Affiliations:** 1 Department of Medicine, Dr. Kiran C. Patel College of Osteopathic Medicine, Nova Southeastern University, Clearwater, USA; 2 Department of Internal Medicine, Lakeland Regional Medical Center, Lakeland, USA

**Keywords:** bone metastasis, brain metastasis, discordant histology, dual primary breast cancer, invasive ductal carcinoma, invasive lobular carcinoma, pulmonary metastases, synchronous bilateral breast cancer

## Abstract

Synchronous bilateral breast cancers (BBCs) with discordant histology are rare and present unique diagnostic and therapeutic challenges, particularly in the setting of metastatic disease. We report a case of synchronous BBCs with discordant histology in the setting of brain, pulmonary, and osseous metastases, underscoring the importance of comprehensive bilateral evaluation and individualized management strategies. A 59-year-old female patient presented with an enlarging, painful exophytic mass on the left chest wall with a past medical history significant for untreated (Grade 3, ER+/PR-/HER2-) invasive ductal carcinoma (IDC) of the left breast two years earlier. During the current admission, imaging studies and assessments demonstrated invasive lobular carcinoma (ILC; ER+/PR-/HER2 IHC 2+) on the right breast with a right cerebellar mass, significant edema and mass effect, nodules in the middle lobe of the right lung, and an abnormal bone scan. Multidisciplinary consultation was obtained from breast surgery, radiation oncology, and neurosurgery; however, the patient declined surgical intervention and palliative radiation therapy and elected hospice care.

## Introduction

Breast cancer is the most commonly diagnosed malignancy among women worldwide and comprises a heterogeneous group of diseases with distinct histopathologic and molecular characteristics [1]. The two major histological classifications of invasive breast cancer are invasive ductal carcinoma (IDC) and invasive lobular carcinoma (ILC) [1]. IDC accounts for approximately 70%-80% of all invasive breast cancers, while ILC comprises approximately 10% [1]. Although both are invasive breast carcinomas, IDC and ILC are biologically distinct with differing histological features, molecular alterations, management, treatment courses, metastatic behavior, and long-term outcomes [1]. 

Bilateral breast cancer (BBC) is increasingly recognized with improvements in breast cancer screening, earlier detection, advances in treatment, and longer patient life expectancy [2]. BBC is classified as either synchronous or metachronous based on the interval between diagnoses, with a six-month threshold commonly used to distinguish between the two [2]. This distinction is clinically relevant because synchronous and metachronous BBC differ in their epidemiology, management strategies, and prognostic implications [2].

Most BBCs exhibit concordant histopathologic characteristics, with a large retrospective cohort study from the Mayo Clinic reporting a 79.8% concordance rate [3]. Discordant BBCs, in which the tumors differ in histologic or biologic characteristics, are less common and may present additional diagnostic and therapeutic implications [3]. This distinction is particularly important in the metastatic setting, as IDC and ILC demonstrate different patterns of metastatic spread and may have differences in treatment response and therapeutic considerations [1]. Determining the histologic and biologic characteristics of each primary tumor can therefore help guide interpretation of metastatic disease and allow individualized management [1].

## Case presentation

The patient was a 59-year-old woman with a past medical history significant for untreated left breast IDC (ER-positive, PR-negative, HER2-negative, Ki-67 index of 40%) and abnormal-appearing left axillary lymph nodes, originally diagnosed two years prior (Table 1). The patient presented to the emergency department with a complaint of an increasingly painful left chest wall mass that had progressively enlarged over the preceding several months (Figure 1). On physical examination, the mass was exophytic, fungating, and ulcerated with overlying skin involvement. Medical records from her initial diagnosis indicated that the patient had previously declined surgery, chemotherapy, and radiation therapy. The patient reported taking anastrozole for approximately three months following her initial diagnosis, but discontinued treatment after losing insurance coverage.

**Table 1:** Immunohistochemical findings of the patient's bilateral breast carcinomas IDC: invasive ductal carcinoma; ILC: invasive lobular carcinoma

Immunohistochemical marker	Left breast IDC	Right breast ILC
ER	Positive	Positive
PR	Negative	Negative
HER2	Negative	2+
Ki-67	40%	Not reported
GATA3	Not reported	Positive
E-cadherin	Not reported	Negative

**Figure 1:**
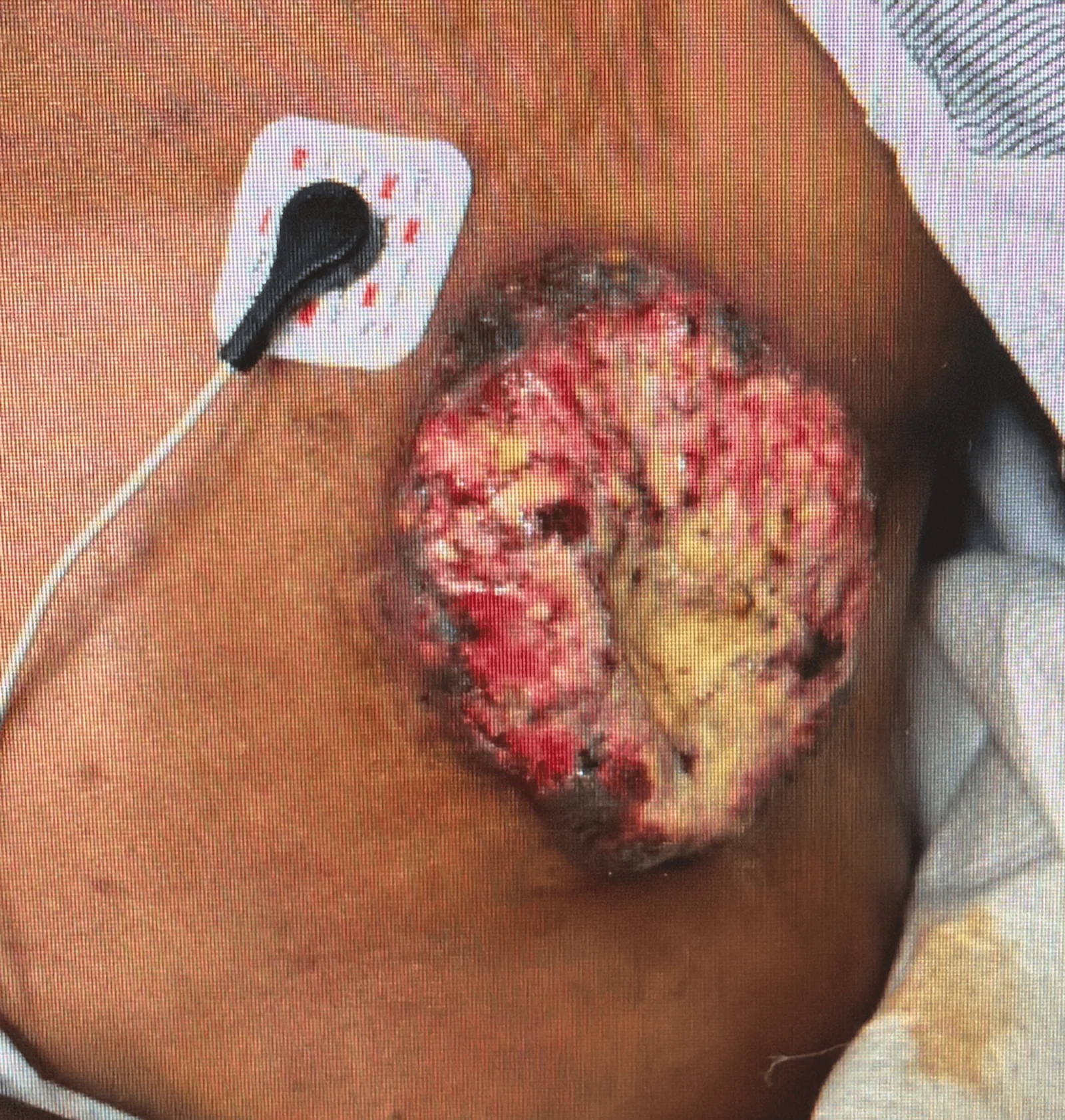
Exophytic and fungating mass involving the left breast

On admission, her vital signs were stable, laboratory findings were unremarkable, and the patient denied any constitutional or cardiopulmonary symptoms. Imaging demonstrated small right middle-lobe pulmonary nodules, enlarged left axillary lymph nodes, an enhancing right cerebellar mass with significant mass effect and possible hydrocephalus, and abnormal uptake on bone scan concerning for osseous metastases. Further evaluation of the right breast was prompted by prominent skin thickening in the right areolar region. Biopsy of the right breast skin revealed a classic type ILC, ER-positive, PR-negative, HER2 IHC 2-positive, GATA3-positive, E-cadherin-negative, with dermal invasion seen in Table 1.

An MRI of the brain with and without contrast and a whole-body bone scan confirmed metastatic disease involving the brain and bone, prompting a multidisciplinary evaluation. Breast surgery determined that no acute surgical intervention was indicated for the breast lesion and recommended outpatient follow-up. Neurosurgery recommended a right suboccipital craniotomy for resection of the cerebellar metastasis, which the patient declined. Radiation oncology also discussed palliative radiation therapy; however, the patient declined and elected to pursue hospice care.

## Discussion

Histologic discordance

Our patient was diagnosed with both IDC and ILC, two main histological categories that exhibit different tumor growth patterns and pathogenesis, resulting in discrepancies in staging [1]. IDC consists of malignant cells that form a cohesive duct-like structure and invade the stroma in a desmoplastic reaction [4]. IDC originates in the mammary ducts with clustered growth and calcifications, typically presenting as a defined firm nodule [5]. In contrast, ILC presents with a hallmark single-file pattern of infiltration consisting of discohesive tumor cells that thread into the stroma due to loss of E-cadherin expression, most commonly driven by CDH1 gene inactivation [1]. ILC originates in milk-producing lobules and often lacks a palpable mass, making it more difficult to detect in later stages [5]. ILC typically presents larger in size at the time of diagnosis with more lymph node involvement [6]. These fundamental differences in cell adhesion underlie the distinct growth patterns, metastatic potential, and management challenges, further underscoring the importance of independent evaluation of each breast lesion, as demonstrated in this present case [7]. 

Impact of histology on treatment selection

BBC with heterogeneous histologies requires treatment decisions that are individualized according to the stage, receptor profile, and histologic subtype of each primary tumor [1]. Although treatment decisions are primarily guided by tumor stage and receptor status, the histologic subtype also influences surgical planning and response to systemic therapy [1]. Compared with IDC, ILC is diagnosed at a larger tumor size and is associated with higher rates of positive surgical margins and mastectomy. Among patients with ER-positive disease, mastectomy rates have been reported to be higher in ILC than in IDC (57% vs. 46%) [8]. Additionally, patients with ILC who receive neoadjuvant therapy have been reported to have higher rates of positive surgical margins at subsequent surgery compared with patients without ILC (21.2% vs. 7.9%) [9]. 

Furthermore, ILC demonstrates lower responsiveness to neoadjuvant chemotherapy than IDC [1]. A meta-analysis of more than 87,000 participants found that those with ILC were less likely to achieve a pathological complete response (pCR) to neoadjuvant chemotherapy than IDC [10]. Axillary pCR rates were also lower in ILC than in IDC (13.4% vs 23.6%) [10]. In contrast, IDC tends to demonstrate better responsiveness to neoadjuvant chemotherapy, particularly in HER2-positive or triple-negative breast cancer [11]. These differences in treatment response may influence surgical planning and underscore the importance of individualized treatment strategies based on tumor biology and histologic subtype [10]. In patients with metastatic disease, treatment should likewise be guided by the biologic characteristics of the metastatic lesions whenever feasible [1]. 

Metastatic patterns and tumor of origin

In patients with discordant BBCs with metastatic disease, determining the likely primary tumor responsible for metastasis can help inform treatment decisions [1]. Common patterns assist in this differentiation; ILC tumors tend to metastasize to the peritoneum, uterus, bones, meninges, and the bone marrow [12]. In comparison, IDC tumors tend to metastasize to regional lymph nodes, lungs, liver, and the bones [13]. In our patient, metastases to the cerebellum, lungs, and bones involved sites commonly associated with both IDC and ILC, making identification of the tumor of origin challenging. Biopsy of a metastatic lesion, when clinically feasible, could aid in determining the tumor of origin, reassessing pathology and ER/PR/HER2 receptor status, and ultimately guiding systemic therapy based on the receptor profile of the predominant metastatic disease [14]. 

Receptor discordance

In patients with metastatic breast cancer, characterization of the receptor profile of metastatic lesions, in conjunction with the primary tumor pathology, is essential for guiding treatment selection [14]. However, receptor discordance between primary breast tumors and their corresponding metastatic lesions has been well documented. A meta-analysis study conducted by Aurilio et al. reported a high level of heterogeneity between primary and metastatic tumors, with a rate of discordance of 20% for ER, 33% for PgR, and 8% for HER2 receptor profiles [15]. In a multicenter study conducted by Hulsbergen et al., discordance was observed in 36.3% of patients between the primary breast cancer and brain metastases, with 22.8% having different subtypes of either PR-positive or ER-positive [16]. Additionally, this study reported that 14.8% of patients with HER2-negative breast cancer had HER2-positive brain metastases [16].

Long-term management and surveillance

Patients with ILC are observed to have a better breast cancer-specific survival rate (BCSS) in the first five years (hazard ratio (HR)= 0.71) in comparison to patients with IDC [17]. However, their BCSS decreases in later years (HR= 1.30 years 6-10; HR= 2.17 years 16-20) [17]. In comparison, IDC had a peak recurrence within the first five years after diagnosis and then declined progressively [17]. According to the National Comprehensive Cancer Network (NCCN), long-term management of both ILC and IDC after primary treatment involves surveillance via physical exam one to four times per year for five years, then annually after five years, and mammography every 12 months after six months of receiving primary treatment [14]. Nevertheless, cancers can go undetected, such as ILC, which can recur with a low chance of survival after five years post-diagnosis, even though there was a good chance of survival within the first five years, suggesting an unknown mechanism that increases ILC potency over time [17]. Hence, clinical providers need to remain alert for metastatic disease and recurrent breast cancer findings in patients, especially if the patient has an ILC [5]. 

Given the potential for late recurrence in ILC, extended adjuvant endocrine therapy may be considered, particularly for patients with hormone receptor-positive disease [18]. In a study by Dixon et al. of 61 patients with 63 ER-positive ILCs treated with neoadjuvant letrozole for more than three months, mean tumor volume decreased by 66% based on clinical assessment and by 54% based on mammographic assessment after three months of treatment [18]. Furthermore, extended endocrine therapy with an aromatase inhibitor for an additional three to five years after an initial five years of tamoxifen therapy has been shown to provide benefit in selected patients, particularly those with lymph node involvement [19]. 

Clinical significance

Although synchronous BBCs with discordant pathologies are uncommon, the coexistence of multiple metastatic sites represents an even rarer and more clinically complex presentation [1]. The presence of metastatic disease further complicates the diagnostic evaluation and management of discordant BBCs, particularly when the primary tumor responsible for metastatic spread cannot be identified [14]. In the present case, the tumor of origin for the brain, bone, and lung metastases could not be determined. Had the patient elected treatment, biopsy of a metastatic lesion, when clinically feasible, together with assessment of its receptor status, would have been valuable for identifying the tumor of origin, confirming the receptor profile of the metastatic disease, and guiding individualized systemic therapy.

## Conclusions

This case highlights the rare occurrence of BBCs with discordant histologies and underscores the importance of independent pathologic evaluation and management of each breast lesion. This patient’s presentation with brain and bone metastases further emphasizes the aggressive and heterogeneous nature of advanced breast cancers and the importance of timely multidisciplinary management. Although synchronous BBCs are relatively uncommon, recognition of their potential for biologic discordance is essential to avoid assumptions of tumor homogeneity and to optimize treatment planning. Continued reporting of similar cases may further refine current understanding of the diagnosis, treatment, and long-term management of these rare and biologically heterogeneous malignancies.
